# Capturing Interactive Occupation and Social Engagement in a Residential Dementia and Mental Health Setting Using Quantitative and Narrative Data

**DOI:** 10.3390/geriatrics1030015

**Published:** 2016-06-28

**Authors:** Mark Morgan-Brown, Joan Brangan

**Affiliations:** 1 Occupational Therapy Department, Mental Health Services, Cavan General Hospital, Lisdarn, Cavan H12 N889, Ireland; 2 Discipline of Occupational Therapy, Trinity College, Saint James’s Hospital, James’s Street, Dublin D08 RT2X, Ireland; jbrangan@tcd.ie

**Keywords:** social engagement, interactive occupation, elderly, mental health, dementia, residents, staff, residential, nursing home, environment

## Abstract

Objectives: Despite an abundance of research acknowledging the value of interactive occupation and social engagement for older people, and the limits to these imposed by many residential settings, there is a lack of research which measures and analyzes these concepts. This research provides a method for measuring, analysing and monitoring interactive occupation and social engagement levels of residents in a secure residential setting for older people with mental health problems and dementia. It proposes suggestions for changes to improve the well-being of residents in residential settings. Method: In this case study design, the Assessment Tool for Occupational and Social Engagement (ATOSE) provided a ‘whole room’ time sampling technique to observe resident and staff interactive occupation and social engagement within the communal sitting room over a five-week period. Researchers made contemporaneous notes to supplement the ATOSE data and to contextualise the observations. Results: Residents in the sitting room were passive, sedentary, and unengaged for 82.73% of their time. Staff, who were busy and active 98.84% of their time in the sitting room, spent 43.39% of this time in activities which did not directly engage the residents. The physical, social and occupational environments did not support interactive occupation or social engagement. Conclusions: The ATOSE assessment tool, in combination with narrative data, provides a clear measurement and analysis of interactive occupation and social engagement in this and other residential settings. Suggestions for change include a focus on the physical, social, occupational, and sensory environments and the culture of care throughout the organization.

## 1. Introduction

If changes are introduced in residential settings, improvements and positive outcomes are difficult to track when trying to study the effectiveness of these changes. There is a need to be able to use interaction and engagement as quantitative outcome measures in order to define the success or failure of environments and environmental change. Using a case study of older people with dementia and other mental health problems in a secure residential setting, this research describes a method of benchmarking, analyzing, and monitoring levels of social engagement and interactive occupation. It examines environmental and staffing factors influencing resident behaviours, and offers solutions for change which are applicable to a broad range of residential settings for older people. 

### 1.1. Theoretical Framework

This research is guided by Activity Theory [1] and Continuity Theory [2]. Activity theory states that the well-being and life satisfaction of older people is dependent upon maintaining social connection and maintaining roles and activities which affirm one’s self-concept [3]. It maintains that activity patterns and personal values create a rich and satisfying life, happiness, and better chances of success with later tasks [3,4]. Greater meaningful activity participation is associated with better psychological well-being and health-related quality of life [5]. Continuity Theory elaborates on Activity Theory and emphasizes the crucial role that continuity of activity and social patterns has in preserving a sense of core identity and sense of self [4,6].

### 1.2. Social Justice and Occupational Justice

Older people continue to develop as individuals even when their physical, mental and cognitive abilities decline. However, there is a tendency to define the ageing population, especially those in congregate settings, in terms of their disability, risks, safety, and medical care needs, and to overlook their psychological needs, including their needs for participation in leisure [7,8]. Equal importance is not given to their requirement for useful and meaningful occupational engagement, social connection, maintaining function, and fulfilment [9,10]. This can be construed as an ageist viewpoint, as if elderly people lose their needs for individuality and interests and are not deserving of social and occupational engagement in the same way as younger people are [8].

Most people do not choose to live in communal residential care. Residents with dementia can lose their sense of self, connection, and belonging. They are placed into a foreign environment in which they may lose the dignity of having their own identity [11] and they are expected to conform to alien and unfamiliar daily routines and time schedules. This unfamiliar environment includes strangers, uniforms, language, and commands. Residents can spend their day in isolation and inactivity, with loneliness, boredom, helplessness, and lack of meaning [10,12]. They frequently ‘give up on themselves’, grow apart from familiar relationships, and perceive the monotony of their new daily lives to be suffering, with a perception that the residence is a ‘hospital’ or ‘communal facility’, but not a home [13]. Instead, residents with dementia want a feeling of ownership of where they live, an ability to make decisions about things which influence their life, and to make a difference and feel useful [10]. There is a need to maintain autonomy and personal identity through social engagement and interaction with meaningful occupations and interactions which are personally valued [14,15,16,17,18].

The care routines, institutional routines, and the over-caring assistance of staff frequently seen in traditional environments is disabling. When elderly residents are given unnecessary assistance in a task by well-meaning staff, this increases their sense of task difficulty, decreases their sense of accomplishment and competence, and makes them less able to complete the task on their own [19,20], which becomes a ‘learned helplessness’ [21]. When the typical communication a resident receives is being told what to do, no matter how nicely, this reduces self-confidence and self-esteem and contributes to mental and physical decline [22].

When asked, residents with dementia state that maintaining independence, having something to do and social interaction are the issues most important to their quality of life [10]. The most important thing for people with dementia living in the community is to be active and do as much as they possibly can [23]. Older people living in the community have greater well-being, reduced functional decline, and reduced mortality if they engage in social and productive activities [24]. Engagement in individualised, meaningful, and familiar occupations increases self-efficacy, future expectations, and positive affect, and contributes to enhancement in quality of life for older people in residential care [25]. However, many institutionalised older people are deprived of opportunities for engagement in work, play, and leisure activities [26].

Russell [27] defines social justice as the ‘ability of people to realize their potential in the context of the society in which they live’ and to maximize their potential for growth, health, and happiness (p. 274). Occupational justice is a component of social justice which emphasizes rights, responsibilities, and freedoms allowing people to experience health and well-being through engagement in occupations [28]. In an occupationally just environment, individuals have a right to experience and participate in occupations for health and social inclusion [28]. Health care professionals have an ethical and moral obligation to identify and address injustices in the care environments in which they work [29]. This is particularly important for people with dementia in institutional care who are poorly, or not able, to petition for themselves. 

Residential environments can be improved to provide better environments for people with dementia. Person-centered non-traditional facilities outperform traditional facilities in potential opportunities for staff interactions and environmental engagement [30].

The aim of this article is to present a direct observation method which provides quantitative data focused on actual interaction and engagement levels in communal sitting rooms which can be used for comparative and evaluative purposes. This article also demonstrates how direct observation can be translated into narrative descriptive data. This qualitative data gives depth to the quantitative analysis of the unit’s occupational interaction and social engagement, which can be used to improve the operational, physical, and social aspects of the environment to benefit resident well-being.

## 2. Method

The ATOSE [31] was originally devised for comparing residents with dementia in two different nursing home settings. It was not designed as a diagnostic specific tool and does not seek to capture elements that reflect a particular diagnostic category. Rather, its focus is on measuring the type of activity/occupation and engagement/interaction of all people present in a given environment including staff, visitors and residents. Most environmental assessments focus exclusively on the residents. In contrast, the ATOSE includes the staff and visitors as essential contributors to the ‘life’ of the room and the dynamic of activity within any care environment. It observes and measures staff and visitor behaviours as part of its whole environment approach.

Other standardized assessments such as the RAI MDS 2.0 [32] uses quality indicators to measure functional, medical, cognitive, and psycho-social status in residential settings. The MDS reports on activity pursuit patterns, and identifies resident’s preferences for activity i.e. (cards, crafts, trips, etc.) whether or not the activity is currently available, as opposed to actual participation of residents in activities. It does not use direct observation to measure actual participation and is therefore subject to reporting bias and variation [32]. Other established direct observation assessments have been developed, such as the Activity in Context and Time (ACT) [33] and Dementia Care Mapping (DCM) [34] which determine actual participation and engagement levels. However, their observations focus on one individual resident at a time. These observations are then summed together to provide an environmental assessment. In contrast, the ATOSE observes residents, visitors, and staff in the whole communal living room in order to give an overview of the amount of interactive occupation and social engagement, or the ‘life’ within this room at each ‘snapshot’ moment in time. The purpose of the ATOSE is to generate a composite evaluation of the whole (physical, social, operational) environment and to use narrative data gathered during the observations to analyse forces which appear to contribute to these findings.

Observational assessments are often used to give the observer’s interpretation of the resident’s emotional state (DCM) and quality of life (ACT). Their focus is primarily on the person centred care within a residential unit. The quantitative focus of the ATOSE has been resolutely kept on observable behaviours and interactions, without the need for the challenging and problematical interpretation and speculation of the emotional states of others and signs of their well-being.

The unit being observed in this study was an approved mental health facility for the elderly. The unit was on the grounds of a large psychiatric hospital which had discharged its other residents into the community over the preceding decades. Some residents on the unit were those patients with behavioural problems which prevented nursing homes or hostels from accepting the patients. Other residents were individuals who had been in nursing homes but the nursing homes were unable to manage them or they were a danger to other residents. There were ongoing discussions within the Health Authority about major redevelopment of the facility and making changes in the operating and social culture of the unit. The purpose of these sets of ATOSE observations was to establish a baseline of interactive and engaged behaviour against which any future developments could be compared.

Irish ethical permission complying with the Declaration of Helsinki (1975/2008) was granted by the HSE North East Area Research Ethics Committee in May 2015 and by the Faculty Research Ethics Committee of Trinity College Dublin in June 2015. Data was gathered on all persons present (residents and staff) in the resident’s communal sitting room during the data collection periods. The observational study recommendations of Van Haitsma et al. [35] were followed—using the same times each day (10:00–12:00 and 14:00–16:00) and observing the same physical space. The researcher(s) sat apart from the residents at a dining table where the whole room could be surveyed, using the two main strategies of observational studies - habituation and minimal interaction [36]. Process consent [37,38] was obtained from the residents, with information tailored to each resident. The observer was prepared to stop at any sign of discomfort or disagreement. Formal signed consent was obtained from the staff. 

The room was observed Monday through Friday, with each observation day falling on a different week over a period of five weeks in order to eliminate any effects which may have been unduly influenced during a particular week. The researcher sat in a corner of the room and scanned the room every five minutes in a clockwise direction, making a tick on the ATOSE sheet in the relevant category the first time each person came into the view of the observer. The social engagement and interactive occupation levels of every person present was marked only once for each five minute ‘snapshot’, as if a series of photographs had been taken. For each two-hour assessment period, there were 24 ‘snapshot’ observations of the whole room.

During the observation periods, the researchers made notes to supplement the ATOSE data and to provide rich examples of the context of observations. These narrative descriptions gave insight [39] into the experience of the residents during the observation period. Another researcher JB observed the room and recorded separately to the main observer (MMB) for one two-hour afternoon session in order to determine the level of agreement (reliability) of the ATOSE. Agreement (inter-rater reliability) between the two researchers was 91.8%. Where there was a discrepancy between the two observers the data from the main observer (MMB) was used in the statistical analysis. Visitor data was collected but is not discussed here as there was only one visitor during the observation days and so the singular data was considered too restricted to be reliable.

## 3. Results

### 3.1. Demographical Information

Table 1 gives a profile of the age, diagnosis, communications skills, and activities of daily living (ADL) abilities of the 20 residents on the unit. The residents were equally divided between male and female. Residents were categorized according to the initial diagnosis they received when they were admitted to the unit. However, staff informally reported that most residents also suffered from cognitive impairment or dementia. Almost half of the residents were able to initiate conversation, with another six able to respond. However, five residents had severe language impairment. The table identifies the dependency levels of the residents with 19 residents requiring assistance with personal hygiene, and 15 residents requiring assistance to get dressed. In contrast, most residents were independent in eating and toileting. During the observation hours there were two multi-task attendants (health care assistants) and four qualified mental health nurses.

### 3.2. ATOSE Quantitative Observational Data Results

Residents spent 36.61% of their time sitting passively staring into space and 40.25% of their time sitting with their eyes closed (40.25%). In total, residents did not engage in any social interaction or interactive occupation for 82.73% of the time they were in the sitting room (Table 2). Residents spent 17.27% of their time in social engagement, interactive occupation (doing something on their own or with others) or receiving interactive care from staff.

Staff were present in the room for 85.83% of all snapshot observations. When in the room, staff spent nearly all of their time being busy and active (Table 3). Only 1.16% of staff time was spent without some interaction with their environment or other people. 28.31% of their time was spent in professional and domestic tasks such as writing notes, setting tables and setting up the medication trolley, 22.04% in conversation with other staff or with residents and 15.78% providing care to residents (such as assisting to mobilize, handing a drink, and adjusting clothes). Overall staff spent 56.61% of their time in interactive behaviours (such as care tasks, shared magazine reading, social conversation) and 43.39% of their time in activities which did not directly engage the residents. There was only one staff member in the room for 42.50% of all observations.

### 3.3. Qualitative (Narrative) Observational Data Results

#### 3.3.1. Social Environment

The almost exclusively female staff showed many examples of great kindness and compassion. For example, when one resident knocked over a glass of juice, a staff member mopped up the spill and replaced it with gentleness and without any negative comment or censure. If staff were involved in a care task, they spoke to the residents to explain what they were doing or to ask if they needed assistance and this was always done in a helpful and calm manner. Otherwise, there was very little conversation and interaction between staff and residents. 

However, staff did occasionally banter with one particular female resident. She attracted this unique attention because she always responded with an animated face and a strong and hearty voice. She was always placed in a central location in the circle of residents, which was convenient for staff to engage with her. On several days, she had a magazine on the table attached to her chair. This simple social tool facilitated conversation as staff were able to point at pictures and turn the pages of her magazine.

#### 3.3.2. Physical Environment

Care tasks frequently took staff out of the sitting room or the staff came into the room and conducted residents away to do some personal care task with them. The distances were great in the sitting room and also in the rest of the building with its long empty corridors. As a result, there were long periods of time with no staff in the main sitting room. 

The room itself was impersonal and unstimulating. There was no softness, warmth, variety, and distinctiveness in the colours, textures, and sounds as in a welcoming domestic home. Unlike a living room at home, the room was lacking familiar personal items to provide conversation prompts. 

#### 3.3.3. Fixed Routines

The institutional environment was defined by the set routines which facilitated staff tasks. The ‘time guillotine’ [40] of meal times were forced by the scheduled arrival of the food from the central kitchens. There was an upsurge in activity levels in the room when staff came into the main room, laid the tables and assisted the residents to go to the tables to await the arrival of food. 

#### 3.3.4. Sensory Environment

As is common in traditional units, the residents spent the day grouped in a semi-circle around the television [8,41]. People with dementia lose interest in television early in the disease and it quickly becomes irrelevant [42]. During these observations, only two men showed any interest. Their visual attention was minimally held for a few moments and then they ignored it. All residents were subjected to the omnipresent sounds from the television programmes, which sometimes included high emotional content. If the TV was not on, a radio played unfamiliar music or broadcast talk shows. There were no signs observed of any interest of the residents in the radio.

#### 3.3.5. Interactive and Occupational Environment

As mealtimes drew closer, residents became noticeably energized and staff came in and out of the room with greater frequency to undertake domestic catering tasks or take residents out for personal care before the mealtime. The open plan of the room meant that some residents saw the tables were being set and got themselves ready to go to the tables.

One male resident took out the rubbish before lunch on a daily basis. He was given the access codes for the locked doors and undertook his chore with a sense of responsibility, confidence and assurance.

The staff discouraged any occupational activity which could create disruption or contamination and lead to falls. A female resident was observed to adjust table settings, fill glasses with water or juice and bring another resident to sit at one of the tables. On a few occasions she was discouraged by staff from pouring or undertaking other jobs. Despite this, she continued to involve herself in these tasks. She had a companion who usually sat next to her. If she got up, her companion would follow her. Staff occasionally discouraged her from following her friend. As Settersten [43] comments ‘institutions may also regulate relationships in determining which types are permissible or in monitoring how they are experienced’ (p. 219).

As staff never stayed in the room for long, they were unable to implement and follow through structured daily household activities to engage the residents or initiate group activities. In addition, the environment was bare of supplies and equipment which could be used by staff to engage and interact with the residents on an individual basis (such as cards, magazines, puzzles, or household chores).

In a domestic home environment, when someone enters or leaves a room, this generates interest and conversation. Residents showed little or no interest in the comings and goings of others, the interactions of staff with other residents, or staff work tasks, such as the preparation of the medication trolley. Staff, in turn, focused their attention on the task at hand or the resident with whom they were working.

## 4. Discussion

Increased activities and social interaction have a positive effect on physical function, agitation, alertness, passivity, engagement, affect, and mood [44,45,46,47,48,49,50,51]. As a consequence of these and other studies, there is a general consensus that social engagement and activity levels are important contributors of quality of life and well-being for people with dementia in nursing homes. However, the primary emphasis of this journal article is not to describe or promote specific types of social or activity interventions. Instead, the aim of this article is to highlight issues around occupational and social justice in long term residential settings for older residents.

Occupational and social justice theories extend a fundamental right to every person to be interactive and engaged and interactive irrespective of disability. This journal article advocates treating older cognitively dependent and psychologically impaired residents with insight and according to their fundamental humanity. Settersten [43] writes that the craving to be appreciated by others is fundamental for each of us and includes the deep human principles of belonging, compassion, mattering, and purpose. In this setting, vulnerable residents had few occupational and social opportunities to be appreciated, to contribute, and to belong.

Health care research should always endeavour to tell us about the human condition, to create knowledge about the realization of human potential, and to promote social justice in a real world situation [27]. The purpose of this article is to assist in the problem solving of the challenge of occupational and social justice in such environments. This research will discuss the insights from the observational process. Issues which impact on interactive occupation and social engagement identified in this environment are discussed below.

### 4.1. Staff Training

Almost all the social interaction was between the staff and residents and usually task related. ‘Communicative collapse’ is the phenomenon of residents seeming to be unable to have conversations with others, so dialogue is entirely dependent upon staff facilitation [21]. A specific example was when a staff member was able to encourage a male resident to go for a walk. After several attempts, the resident finally understood and he appeared delighted to join the staff for the walk. Upon returning, the staff member outpaced the resident when coming back into the room, as the task was accomplished and as the conversation was exhausted. The resident followed and stood behind the staff member for an extended period of time, looking as if he wanted the interaction to continue, but not being able to initiate the conversation himself. This is in contrast to the female resident (with the magazine) who had more language and social skills and where the magazine could be used as a prompt for conversation. Staff training is required in how to stimulate and maintain conversation. The social engagement was hampered by the stimulus poor impoverished environment. Staff interaction is improved by using enjoyable and spontaneous activities to engage the residents. Furthermore, effective communication by staff decreases aggression and depression and increases quality of life for residents [22]. In this study by Sprangers, Dijkstra and Romijn-Luijten, nursing aides were trained to use short instructions, positive speech and biographical statements during personal care. This had a positive effect on residents, as well as being beneficial for staff, by reducing stress and caregiver burden.

### 4.2. Sensory Environments

Environmental sensory input is rarely considered. Noise, lighting, and ambient temperatures are associated with reduced social interaction, increased stress hormones, negative mood, and lower quality of life in residents with severe dementia [52]. The physical environment (music, homelike environment, functional modifications, small-scale group living, light, multi-sensory stimulation, and building footprint) all have some effect on the physical activity for people with dementia in a residential environment [53].

The relentless noise of the TV and/or radio programmes appeared to mask silence, and inactivity. The residents were observed to show negligible interest in the TV. Setting residents in front of a TV without knowing whether or not this has meaning for them undermines dignity [8]. Loud, aggressive, and disturbing noises overwhelm and immobilize residents [54].

The staff worked within an austere environment. The long hallways to the bedroom where personal care was carried out, meant there was a disengagement between these two environments, rather than being connected into daily life. These distances stretched much further than the distances within an ordinary house. The institutional environment and task focused work were reinforced by most of the beds lined up in rows on a male and female ward. An institutional environment reinforces the institutional nature of work. This in turn reinforces the objectification of the residents as being someone with a ‘diagnosis’ which requires being cared for rather than an individual who has a life which is ‘worth living’.

### 4.3. Recommendations for Change

#### 4.3.1. Environments Supporting Engagement

A striking feature of this data is that staff spent only 1.16% of their time in the ‘no interaction’ category. In contrast, residents spent 82.73% of their time without purposeful interaction or engagement with others. Occupational patterns of the room were defined by busy staff and passive sedentary non-engaged residents who were frequently sleeping. This has been observed in other research [6,41,54,55].

Traditional environments are typified by impoverished environments where the residents sit in a large circle sleeping or staring into space, lacking in spontaneous conversation and interaction, passively waiting for staff to stimulate and engage in conversation or activity [21,41,55]. In contrast, in their own homes, people are generally doing something when they are in their sitting rooms. It is not normal for people to live together and not acknowledge or speak to each other. It is harmful to mental and physical health to sit in a room and do nothing or sleep most of the day. Admission to institutional care has consequences for residents. Newly admitted residents who become dissatisfied with their daily life occupations in the nursing home are more at risk of an early death [56]. This stark fact reinforces the importance of personal independence, homelike routines and involvement in over-learned familiar tasks, which reinforce residents’ sense of personal continuity as a mother, a farmer, a homemaker, etc. These tasks also give conversation topics. Social engagement is correlated to longer survival in long-stay nursing home residents [57]. Spontaneous interactions give a sense of ‘aliveness’ to conversations and interaction, making them much more meaningful than stereotypical care task conversations [58]. They are more personal and more life-enhancing than those interactions which are based solely around care tasks and fixed routines initiated by staff.

Undertaking household tasks gives residents a chance to belong through active participation and contribution to the household, and a sense of involvement, responsibility, and meaning. For a woman with dementia who has limited new learning ability, opportunity to preserve these ‘ingrained’ household skills can be vital. Creative groups enhance staff attitudes and stimulate more and better quality staff interactions with residents, as well as more engagement from the residents [59]. Facilities need to be designed to give opportunity to watch others [58], as well as allowing milling around, walking and, thereby, spontaneous and chance encounters [54]. Pets, animals, and children provide interest, spontaneity, as well as social and occupational interaction and have been shown to decrease physical, behavioural, and emotional symptoms [12,51,60,61].

Half the residential population of this unit was male. Except for the examples mentioned previously, the men in this unit were not engaged. It is more difficult to engage older, dependent male residents, as most residential occupations and activities do not relate to a male sense of core identity and sense of self [62]. Work related activities are generally preferred over purely manipulative activities [16]. In this unit, taking out the rubbish was a congruent fit for one male and involved him in a contributing household task.

#### 4.3.2. Provision of Specialized Staff to Provide Occupational Interaction and Social Engagement

In institutional settings, the medical and physical needs of the residents tend to be prioritised over their needs for connection and engagement [8]. Institutional care is defined by its focus on the human body, but not the human being [63]. Residents can be clean and well fed but be unstimulated, inactive, and socially excluded [64]. When staff in this study were not present, there was little or no activity or social communication in the communal sitting area, as is frequently seen in other residential environments [40,65].

There is a need for staff to adapt daily activities to encourage resident participation in what is now their own home. In this setting, the majority of staff interactions with residents were brief and linked to undertaking care tasks. A lack of therapeutic activities points to the values and beliefs of the care and nursing staff [64]. In contrast, there are specialist staff who define the success of their work, not by personal care given, but by the interaction and engagement of residents [21] and these staff include occupational therapists, occupational therapy assistants and activity therapists. Employing such staff, or redefining an existing staff role, would be a specific measure to ensure more social and occupational interaction for residents. This reorientation of operational approach requires managerial direction, as well as funding and organisational support. Encouragingly, even small interventions can make a difference. For example, a weekly occupational therapy group focusing on familiar physical activities and cooking created improvements in cognitive function, physical health, depression and quality of life [66].

Staff were in the sitting room for most of the snapshot observations. They came into the room to undertake a specific task and then left again. This meant that no individual staff member took responsibility for the environment and its activities over the whole day. Allocating a staff role, such as a homemaker, permanently to the room to monitor and engage the residents is a way of increasing occupation and conversation. A homemaker role defined by kitchen and cooking duties in an open plan room with integral kitchen with altered operational policies allows person-centred choice and significantly increases the interactive occupation and social engagement of residents and staff, improving quality of life for residents, working life for staff, and visiting experience for relatives [40]. 

There was only one visitor during the whole research period. Staff confirmed that visitors were rare. In an ordinary domestic home, visitors are normally offered hospitality to make them feel greeted and welcome. One component of this lack of visitation may be a lack of welcome. This would involve staff being in the room, knowing what to do and having facilities within the room to make the cup of tea. Having a homemaker with access to a kitchen in the sitting room area would enable this welcome.

#### 4.3.3. Physical Design

While the physical designs of environments often receive most attention, the social environment and operational policies are more important than the physical design [67]. The systematic review of Bradshaw, Playford, Riazi [68] found that residents did not define a homelike environment by specific physical features but by: connection with others; participation in meaningful tasks; decision making and choice; staff knowing and respecting each individual’s life history; and non-rushed and non-forced staff routines. 

Environments can include reminiscence areas, multi-sensory rooms and areas which facilitate group and individual sessions [63]. However, such spaces are ‘only as effective as the staff who use them’ p. 655 [69]. 

Household and domestic tasks facilitated by female staff will rarely engage male residents. Half of all residents in this unit were male. Being an older male resident with challenging behaviour and ADL dependency predicts a poor activity involvement, which is not overcome by being in a more homelike environment [62]. Heavier outdoor gardening, care of animals, and farm yard work are used on the Green Care farms for dementia [70,71]. Whear et al. [72] provide a systematic review of outdoor spaces beneficial for agitation. In the context of the type of unit under study in this article, aspects of plant based and animal care tasks can be done in a suitable indoor environment [51].

#### 4.3.4. Social Policies

It is difficult for staff to initiate and prolong social interaction with residents with dementia [21]. However, simple, inexpensive changes in institutional practices create highly significant improvements in the amount and type of communication. Communications skills training improves the lives of the residents, and enables staff to feel better about the job and be less distressed [22]. However, an organisational strategy is crucial to establish formal monitoring and management feedback, staff incentives, and self-monitoring mechanisms, or the communications strategies will fade over time [22,59,73,74].

#### 4.3.5. Operational Policies

Good muscle strength and balance are correlated with better quality of life for people with dementia in residential care [75]. Physiotherapy sessions providing a high intensity functional exercise programme twice a week statistically improved lower limb strength and balance, as well as reducing agitation and behavioural problems [48]. Many gains were maintained for three months post intervention, reducing the need for medication to manage behavioural symptoms. Other studies show the effectiveness of professionally devised exercise programmes on balance, gait, cognition, attention, and quality of life [75,76].

In this study, the residents depended upon the staff for interaction and engagement. Otherwise, they remained in their chairs, staring into space or with their eyes closed, appearing inactive and disengaged even when other things were happening in the room. The challenge is to create an environment where residents do not act as ‘guests’ [21], but are involved and participate, taking ‘ownership’ of their home environment and feeling useful [10]. A meaningful environment for people has participation, occupation, and spontaneous social engagement. Resident autonomy and choice defines person-centred care and requires an environment designed to allow flexibility in the major time guillotines around mealtimes, as well as staff dedicated to facilitate this choice [40].

Written care plans should include specific professionally assessed occupational and social interactions from staff which are prioritised in the way that personal care is prioritised. These daily minimum interventions should be assessed by a trained professional to determine the appropriate interventions. For example, it could be a verbal discussion around sport with one person. For a non-verbal client it could be a hand massage or other tactile interaction. The environment should set a minimum level of guaranteed participation and engagement in daily life roles.

### 4.4. Limitations

This observational assessment was purposefully restricted to the main sitting room for two hours morning and afternoon, so all observations and discussions relate only to these observations. Staff interactions with residents, for example, providing personal care in the bedrooms, toilet, and shower areas away from the main sitting room, were not assessed by the ATOSE and are, therefore, not included in this study.

The assessment process does not assess quality of life per se or emotional states, but purposefully focuses on the interactive occupation, social engagement and the non-interaction and non-engagement of all persons present in the room.

The ATOSE itself does not place a value on interactions, or suggest that certain interactions are better than others. However, sitting in an environment with a specific task and then observing and making sense of what is happening in the room gives a unique opportunity for in-depth contemplation. These insights have been given as narrative data.

## 5. Conclusions

There has been widespread criticism of traditional models of residential care for older people and people with dementia in the research literature. Older residents with cognitive impairment who move into a long term care environment are at risk of physical, cognitive, mental, and functional decline caused by their environment [53,56,57].

Recommendations for change focus on the physical, social, and sensory environments as well as change in the culture of care throughout an organization [77]. Occupational and psychosocial interventions reduce the number and severity of neuropsychiatric symptoms and enhance physical ability and general well-being [78].

A willingness to address the occupational interaction and social engagement needs of residents can minimize occupational and social deprivation. However, this requires determination, resources, and imagination to allocate staff time, provide training, and create a new physical environment with flexible routines, spontaneity, choice, and interaction [40,77]. Changes need to be sustainable which require feedback schedules, accountability, and review.

This research demonstrates a method for capturing quantitative and narrative evidence of levels of occupational and social engagement to objectively evaluate residential care environments. This can be used as a baseline to compare the effectiveness of physical, operational, and social environment change, using daily interactive occupation and social engagement as the operative outcome measures [77].

## Figures and Tables

**Table 1:** Profile of Residents.

Age	n	Diagnosis	n	Communication Ability	n	ADL Dependency	n
60–70	7	Dementia	4	Able to initiate conversation	9	Eating	4
70–80	8	Bipolar	4	Will respond but not initiate	6	Dressing	15
80–90	3	Psychosis	7	Greatly reduced conversation	1	Hygiene	19
90+	2	Depression; anxiety	2	No real conversation	4	Toileting	5
		Intellectual disability	3				

**Table 2:** Residents Engagement and Interaction Levels.

Resident: Engagement and Interaction Levels	Examples of Behaviours	% age time spent
Social Engagement	Talking with another, non-verbal interaction (e.g., patting another on the hands)	3.72
Interactive Occupation	Active reading, participation in individual or group activity, walking, drinking, eating	10.70
Receiving Care	Medication, adjusting clothes, re-positioning	2.85
Subtotal: Engagement and Interactive Occupation		17.27
No Interaction	Calm sitting, staring into space, disassociated from the environment	36.61
Eyes Closed	Dozing, sleeping, disconnected from the environment with closed eyes	40.25
Agitated	Forceful rocking, distressed verbal noises	0.5
Self-Stimulatory	Repetitive scratching, repetitive touching	5.37
Subtotal: Non-engaged and non-Interactive		82.73
Total of all Categories		100 %

**Table 3:** Staff Engagement and Interaction Levels.

Staff: Engagement and Interaction Levels	Examples of Behaviours	% age time spent
Social Engagement	Talking with other staff, residents, visitors	22.04
Interactive Occupation	Undertaking activity with residents such as games, crafts, magazine reading	8.79
Providing Care	Adjusting clothes, transfers, escorting to toilet	15.78
Subtotal: Interpersonal Engagement		56.61
No Interaction	No active involvement	1.16
Catering Tasks	Setting tables, preparing food without involvement of residents	10.44
Domestic Tasks	Sweeping, cleaning without involvement of residents	3.48
Professional Tasks	Medication trolley, telephone, hand-over discussions	28.31
Subtotal: Interpersonal non-Engagement		43.39
Total of all Categories		100 %
